# The cellular composition of the lung lining fluid gradually changes from bronchus to alveolus

**DOI:** 10.1186/s12931-021-01882-x

**Published:** 2021-11-04

**Authors:** S. D. Pouwels, Janette K. Burgess, Erik Verschuuren, Dirk-Jan Slebos

**Affiliations:** 1 Department of Pathology and Medical Biology, University Medical Center Groningen, University of Groningen, Hanzeplein 1, 9713 GZ Groningen, The Netherlands; 2 Department of Pulmonary Diseases, University Medical Center Groningen, University of Groningen, Groningen, The Netherlands; 3 Groningen Research Institute for Asthma and COPD, University Medical Center Groningen, Groningen, The Netherlands

**Keywords:** Bronchoalveolar lavage fluid, Epithelial lining fluid, Neutrophils

## Abstract

Although large advances have recently been made mapping out the cellular composition of lung tissue using single cell sequencing, the composition and distribution of the cellular elements within the lining fluid of the lung has not been extensively studied. Here, we assessed the cellular composition of the lung lining fluid by performing a differential cell analysis on bronchoalveolar lavage fluid (BALF) and epithelial lining fluid (ELF) at four different locations within the lung in post-lung transplantation patients. The percentage of neutrophils and lymphocytes is reduced in more distal regions of the lungs, while the percentage of macrophages increases in these more distal regions. These data provide valuable information to determine which lung lining fluid sampling technique and location is best to use for measuring specific factors and biomarkers, and to increase the understanding of different cell populations in specific lung regions.

**To the editor:**

With the recent advances in single cell sequencing techniques, the overall profile and distribution of cells present within the lungs, both in health and disease, have been largely mapped out [1]. However, limited data is available about the cellular composition of the lining fluid throughout the bronchial tree. Already in 2007 a series of review articles described the potency of using bronchial alveolar lavage fluid (BALF) both as a research and a diagnostic tool [2, 3]. Subsequently, it has been recognized that BALF cell distribution data can be used to diagnose various diseases, e.g. lung cancer, pneumonia, diffuse lung disease, interstitial lung diseases and acute and chronic allograft rejection post lung transplantation [4–7]. It is well-described that in healthy individuals more than 80% of the cells found in BALF are alveolar macrophages, around 5–15% are lymphocytes and a few percent of the cells are neutrophils, eosinophils or mast cells, and that the amount of neutrophils or eosinophils can rapidly increase in patients with airway inflammation, e.g. COPD or asthma patients [2, 8]. Besides collecting lung lining fluid using a bronchoscopic washing, where a large volume of saline (25–300 mL) is flushed and collected from a specific lung segment, epithelial lining fluid (ELF) can also be collected using bronchoscopic microsampling probes (BMS). BMS are absorbent probes that can collect ELF at a specific site with minimal dilution of the sample, a major difference from BALF collection. It is, however, not possible to collect ELF using BMS at the lower airways because these are bronchoscopically inaccessible. The concentration of soluble factors, e.g. cytokines, measured in ELF collected using BMS, from now on called ELF, are in general higher compared to the concentrations of these factors measured in BALF [9]. Although some studies compare the concentrations of soluble factors in ELF collected at the central and peripheral airways [9, 10], no studies have been performed assessing the cellular composition in BALF or ELF at different locations within the lungs.

Here, we assessed the relative amount of neutrophils, macrophages and lymphocytes in lung lining fluid at four different locations of the lungs, ranging from the main bronchus to the alveolar compartment. To this end, we bronchoscopically collected BALF and ELF from seven lung transplant recipients. Samples were collected during a routine surveillance bronchoscopy on average 19 months (range 4–25 months) after transplantation. None of the patients had chronic lung allograft dysfunction or used azithromycin. Patient details are summarized in Table 1. The study was approved by the medical ethics committee of the University Medical Center Groningen, The Netherlands (METc: 2003017), and all subjects provided written informed consent. ELF was collected using microsampling probes (BC-401C; Olympus, Tokyo, Japan) at two distinct locations, the central airways (after the 1st generation at the main bronchus level) and more peripherally at the 3rd generation at the segmental bronchi. BALF was also collected at two distinct locations, a mini-BALF of 40 mL was performed at the small intra-segmental bronchi (4–15th generation) and a regular BALF of 150 mL was performed reaching the bronchioles and the alveolar compartment (see Fig. 1A). All ELF and BALF samples were centrifuged (5 min, 1000*g*) and differential cell counts were performed by cytospin cell-differentiation. Cytospins were prepared using a cytocentrifuge (Shandon Life Science, Cheshire, UK), and subsequently fixed and stained with Diff-Quick (Dade A. G., Dudingen, Switzerland). Per cytospin 300 cells were identified and differentiated into macrophages, neutrophils, and lymphocytes by standard morphology and staining characteristics.

**Table 1:** Patient characteristics

Age	56 (24–62)
Sex (M/F)	5/2
Smoking status (non-smoker/ex-smoker)	5/2
Reason for tx (PAH/A1AD/UIP/CF)	2/2/2/1
Timing bronchoscopy after tx (months)	19 (4–25)

Age and timing after transplantation shown as median with range, all other data shown as n

*M* male, *F* female, *tx* lung transplantation, *PAH* pulmonary arterial hypertension, *A1AD* Alpha-1 antitrypsin deficiency, *UIP* usual interstitial pneumonia, *CF* cystic fibrosis

**Fig. 1:**
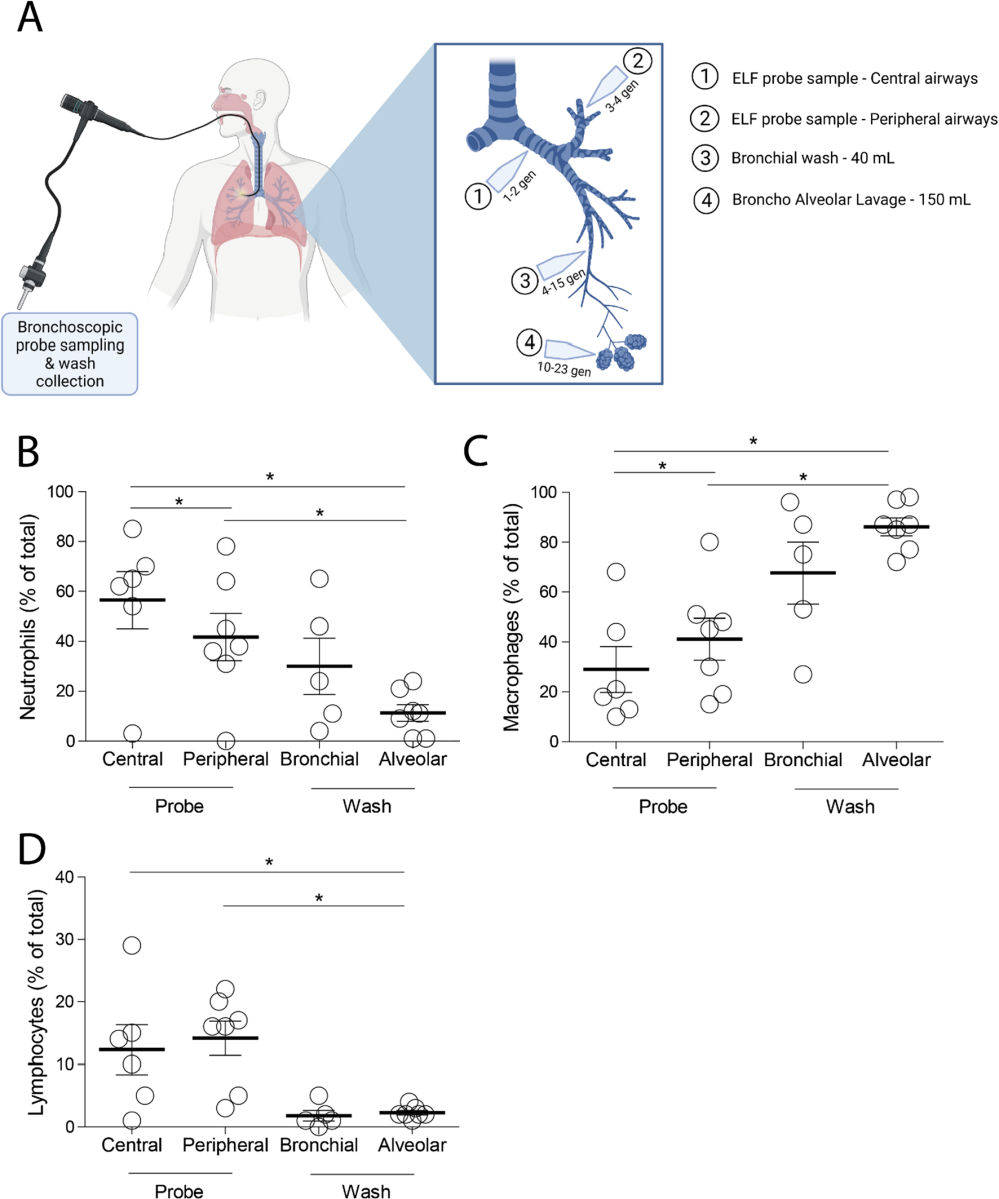
The cellular composition of the lung lining fluid gradually changes depending on the location of sampling. **A** Schematic overview of sample collection sites. Epithelial lining fluid (ELF) samples were bronchoscopically collected using bronchoscopic microsampling (BMS) probes at two different locations, (1) at the main stem bronchus (1–2st generation) and (2) at the large intrasegmental bronchi (3–4th generation). (3) A small bronchial washing (40 mL of saline) is bronchoscopically applied and collected at the small intrasegmental bronchi (4–15th generation) and a (4) broncho-alveolar lavage (150 mL of saline) is bronchoscopically applied and collected at the bronchioles and alveolar compartment. A differential cell count is performed on the collected lung lining fluids and the percentage of **B** neutrophils, **C** macrophages and **D** lymphocytes of the total cell number shown. Data was collected from seven patients, but due to technical issues one central probe sample and two bronchial wash samples were missing. Data is shown as individual data points and mean ± SEM. Statistical significance was tested between samples using a Wilcoxon Signed Rank test, *p < 0.05

Here, we showed that the percentage of neutrophils is significantly lower in ELF samples collected at the peripheral airways compared to ELF collected at the central airways (Fig. 1B). Furthermore, BALF samples contain a lower percentage of neutrophils compared to ELF samples and in general a trend is visible that the percentage of neutrophils decreases when going deeper into the airways (Fig. 1B). When analyzing the percentages of macrophages, the opposite pattern was observed, with significantly higher percentages of macrophages in the peripheral airways compared to the central airways and significantly higher percentages of macrophages in BALF compared to ELF samples (Fig. 1C). The percentage of lymphocytes was significantly lower in BALF samples compared to ELF samples (Fig. 1D). In general, a gradual trend was observed with lower percentages of neutrophils and lymphocytes and a higher percentage of macrophages in the lower airways.

Our study indicates that when using lung lining fluid samples either for diagnostic biomarker purposes or as research tool, the location of sample collection is very important. Therefore the sampling site for the collection of lining fluid should be standardized between all subjects. Furthermore, when measuring biomarkers mainly released by neutrophils or lymphocytes it is advised to use central airway ELF samples collected using a bronchoscopic microsampling probe, while for studying factors released by macrophages a BALF sample from the lower regions of the lungs would be advised.

The samples collected in our study were collected using either a microsampling probe or a washing. To date, no data is available comparing the efficiency of these techniques to collect specific cell-types. It is therefore possible that the used technique affects the percentage of specific cell-types. Furthermore, a potential limitation of this study is the low number of subjects (n = 7) and the large disease heterogeneity of our subjects (Table 1). A larger study is needed to fully investigate the cellular sub-types which can be found at the different locations, potentially using novel techniques such as single cell sequencing.

In conclusion, our study shows that the cellular composition of the lung lining fluid alters when moving more distal in the airways. Deep into the lungs higher percentages of macrophages can be found, while the fluid lining the larger airways mainly contains neutrophils and lymphocytes. These data should be taken into account when deciding which lung lining fluid sampling technique and location should be used to measure specific factors and biomarkers.

## Data Availability

The datasets used and/or analyzed during the current study are available from the corresponding author on reasonable request.
